# Optimal charge scheduling and on-board control of an urban electrified BRT fleet considering synthetic representative driving cycles

**DOI:** 10.1038/s41598-024-55725-y

**Published:** 2024-03-06

**Authors:** Ahmed Ali, Ahmed F. Ayad, Mostafa Asfoor

**Affiliations:** 1 Automotive Engineering Department, Military Technical College, I. Fangari, Cairo, Cairo 11766 Egypt; 2 Branch of Aerospace Engineering, Military Technical College, I. Fangari, Cairo, Cairo 11766 Egypt; 3 National Institute for Advanced Transportation Technology (NIATT), University of Idaho, Moscow, ID 83844 USA

**Keywords:** Electric BRT fleets, Urban electric transportation, Charge scheduling, Synthetic driving cycle, Vehicular control, Intelligent transportation systems, Electrical and electronic engineering, Mechanical engineering

## Abstract

This paper presents a comprehensive approach for optimal charge scheduling and on-board vehicular control of electrified fleets based on synthetic driving cycles. The proposed approach is conducted within a real case-study in Cairo, Egypt, whereto a representative distance-based driving cycle has been synthesized using K-means clustering over a sliding horizon of gathered data-sets. Two multi-objective problems defining optimal charge scheduling and vehicular control have been formulated to achieve minimal energy consumption and operating cost of the fleet . Non-dominant genetic algorithm (NSGA-II) has been implemented to solve the optimization problems jointly considering fluctuating electricity cost of the grid. The comparative evaluation of results reveals an improvement of 19% and 28% in energy consumption and retention of on-board energy accordingly, with less than 2% mitigation of driveability. Moreover, a reduction of 40.8%, 20%, and 21.9% in fleet size, required charging stations, and annual recharging cost respectively has been realized. The main innovation of this work can be put forward as the ability to address the above-mentioned quadrilateral objectives of electrified fleets in a single comprehensive approach, considering synthetic driving cycles and electricity prices to yield a customized-optimal solution.

## Introduction

### Background and previous works

Public transit is considered the backbone of transportation in dense metropolitan cities, where urban traffic demands are incessantly high^1^. Electrified Bus rapid transit (E-BRT) is recognized as an energy-efficient, environmental-friendly, and economic transportation system, that has proven the potential to resolve congestion challenges, and improve the quality of transportation. A significant challenge of E-BRT systems is the long duration of battery charging, which urges the need for optimal power control and rigorous charge scheduling for the fleet^2^. Inept vehicular control and charge scheduling of electrified fleets results in delayed headways trips, long queues at charging station(s), increasing operating cost, and excessive loading of the grid^3^.

These challenges motivated many researchers to tackle the impact of abrupt driving conditions and charging requirements on the optimality of electrified vehicles. In this context, defining representative trip characteristic has been particularly addressed in literature, to yield optimal control strategies for electrified powertrains achieving minimal energy consumption and suitable charging events for each trip.

**Figure 1:**
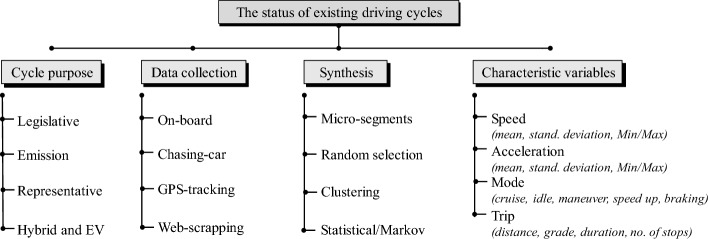
The status of existing driving cycles, considering type and purpose, data acquisition methods, profile synthesis approaches, and characteristic variables^6^.

The important impact of driving cycles on energy consumption and charging requirements of E-fleets attracted many researcher to develop representative speed profiles of commuted trips, including data gathering, processing, analysis, and synthesizing methods of driving cycles^4,5^. The status of existing research on the development of driving cycles can be summarized according to four main perspectives: application and purpose of the driving cycle, data gathering and acquisition methods , synthesizing approach of the time-speed-distance profile , and the considered variables for data clustering and analysis. An illustrative summary of this classification is shown in Fig. 1^6^.

Standard driving cycles have been implemented for many applications including, reconstruction of actual traffic conditions of specific cities/countries for testing procedures^7^, evaluation of electrified powertrains^8^, emission measurements^9^, or legislative testing procedures of new vehicles^10^. Traditional data gathering methods used in driving data collection could be summarized as measurement of trip conditions using on-board instrumentations, chasing-car technique, and hybrid approaches^7^. The use of crowd-sourced datasets from connected global positioning system (GPS) devices is a novel technique that grasps the attention of many researchers increasingly^6,11^.

Construction of representative driving cycles has been conducted in literature using gathering and re-segmentation of micro-trips^12^. Clustering of captured speed profiles has been carried out using different learning techniques, i.e. Markov chains and artificial neural-networks^13^. K-means clustering has been advantageous for driving cycle synthesis due to its computational tractability and attainable accuracy compared to similar methods^14^. Selection of characteristic variables for cluster analysis and synthesis of driving cycles received particular attention in literature considering qualitative aspects (type of variables) and quantitative measures (statistical attributes or each variable)^15^.

The ability to define upcoming driving conditions of traveled route incited the pursuit to investigate multiple strategies that enforce the worthiness of fleet electrification in terms of cost reduction, ride-comfort, and energy efficiency^16^. Preliminary sizing of electric fleets operated at fixed routes has been addressed in literature considering optimal capacity of the on-board energy storage and minimal required number of vehicle in the fleet^17^. Moreover, suitable energy management and vehicular control algorithms have been under development to handle anticipated power demand or upcoming traffic congestions^18^. Optimal scheduling and allocation of charging stops has grasped the attention of many researchers aiming to reduce duration of battery recharging fr the entire fleet and avoid overloading of the grid^19^. Joint optimization of the afore-mentioned objectives has been less frequently addressed in literature to yield an optimal approach towards an energy-efficient operation of E-fleets^20^.

Simultaneous determination of required battery capacity, fleet size, and number of charging terminals has been conducted using deterministic mixed-integer linear mathematical model^21^. Further insights into the inherited uncertainty about power demand of the vehicle has been given using stochastic linear programming in^22,23^. Extended uncertainty about the time-variant cost of electric energy for battery charging has been considered using Markov models in^24^. The approach dealt with upcoming pricing peaks based on akin loading profiles of the grid. Expected charging demand on allocated terminals and entailed stop durations have been minimized using genetic algorithms in^25^.

A novel rolling horizon method to consider the uncertainties in bus operations and to achieve real-time control of bus charging activities which reduced the total charging costs has been presented in^26^. The same concept has been adopted for an optimization model defining optimal charge scheduling, duration, and power, in order to minimize the operational costs of a sustainable charging policy at a university facility^27^. A particular focus on the design of an optimal scheduling scheme has been proposed considering wireless-charging of a bus fleet aiming to minimizing the cost of energy in light of fluctuating electricity prices^28^.

Advanced fast en-route charging and battery swapping has been increasingly considered for daily commuted trips to overcome the limited driving range anxiety and long recharging times during the dwell time^21,29^. Optimal strategies for fast charging have been investigated for a fleet in electric buses in^30^. A threshold for the battery state-of-charge SoC has been defined to minimize the demand to charging facilities by simulating daily charging patterns for the entire fleet. Mixed-integer optimization model has been advantageous for such event-based models, whereby the total charging and operating cost of the electric fleet can be targeted^31^.

### Novelty and contribution

In light of the conducted review and discussion of previous works, following insights into the gaps in research and potential improvements can be put forth:

- First, development of representative driving cycles for commuted routes of electrified fleets is indispensable to define suitable control strategies and charge scheduling of each vehicle. In this context, using crowd-sourced data on traffic conditions has a significant potential to yield accurate speed profiles at mitigated cost of measurement instrumentation and to avoid technical complexity of the data-gathering process. However, accurate analysis of crowd-sourced data that captures relevant clusters of speed profiles has been scarcely addressed in literature.
- Second, control strategies and charge scheduling of electric fleets has a significant potential to minimize infrastructure requirements and operating cost of the fleet. Furthermore, such achievements become more challenging under dynamic pricing of charging energy. In this regard, limited works in literature considered the sequential joint-optimization of the aforementioned objectives for public transportation.

The proposed work in this paper aims to tackle the pointed out gaps in literature and propose a comprehensive approach for optimal fleet sizing, charge scheduling, and on-board vehicular control of public E-fleets. The novelty and contribution of this work can be pointed out as follows:

- optimization of fleet sizing, charge scheduling, and power control has been jointly conducted considering dynamic grid-loading profile and varying electricity prices,
- representative driving cycle for bus routes has been synthesized based on optimal clustering of crowd-sourced traffic data,
- implementation to a real case-study for an electric BRT-fleet for Cairo, Egypt, considering implicit initial and running cost, and
- conducting a comparative evaluation of the achieved results against arbitrary charge scheduling, fleet sizing, and power management strategies.

### Outline

The remainder of this article is organized as follows: modeling of a the electric powertrain and development of the representative driving cycle is presented in “Vehicle model and driving cycle synthesis”. Formulation of the mathematical problem for power management and charge scheduling is given in “Optimal vehicular control and charge scheduling of the BRT fleet”. The analysis of obtained results and relevant discussions are given in “Analysis of results and discussion”, followed by a comprehensive conclusion of the presented work in “Conclusion”.

## Vehicle model and driving cycle synthesis

### Pure electric driveline

The investigated E-fleet in this study comprises a number of mono-sized articulated BRT buses equipped with pure electric drivelines. The propulsion force of the powertrain is delivered by two in-wheel permanent magnet synchronous motors (PMSM) empowered by a pack of lithium-iron phosphate (LFP) batteries. A summary of the driveline and bus characteristics is given in Table 1.

**Table 1:** General specifications of electrified BRT articulated bus.

Sub-component	Specification	Value
Vehicle	Vehicle mass ($m_\text {v}$)	28.5 (Tonnes)
	Frontal area ($A_{\text {f}}$)	8.6 (m^2^)
	Coeff. of rolling res. ($\mu _\text {r}$)	0.009 (–)
	Air density ($\rho _\text {a}$)	1.27 (kg/m^3^)
	Drag coefficient ($c_\text {d}$)	0.3 (–)
	Tire radius	0.477 (m)
Powertrain	Type (–)	PMSM (–)
	Topology (–)	In-wheel (–)
	Rated power (–)	2 × 150 (kW)
	Rated torque (–)	2 × 550 (Nm)
Battery	Type (–)	LFP (–)
	Voltage	600–650 (V)
	Capacity ($Ah_\text {b}$)	315 (kWh)

**Figure 2:**
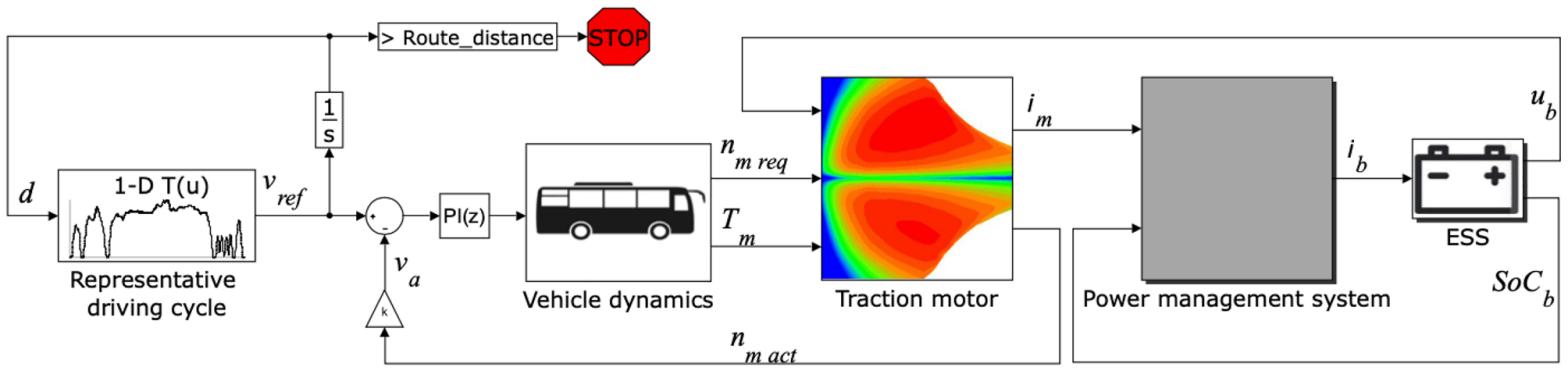
Driveline layout and modeling of electric bus in Matlab/Simulink environment.

Mathematical modeling of the electric powertrain is carried out in MATLAB/Simulink environment considering forward/backward simulation as illustrated in Fig. 2. Retention of the desired speed profile according to considered driving cycles is ensured using a custom proportional-integral (PI)-controller^32^. Required traction forces are calculated backwards based on the desired speed as

1 $$\begin{aligned} F_\text {t}&= \underbrace{m_\text {v} \frac{dv}{dt}}_{\textit{Inertia}} + \underbrace{\frac{A_\text {f} \ \rho _\text {a} \ C_\text {d} \ v^2}{2}}_{\textit{Air drag}} +\underbrace{m_\text {v} \ g\ \sin \theta }_{Grade \ res.} + \underbrace{\mu _\text {r}\ m_\text {v}\ g\ \cos \theta ,}_{Rolling \ res.} \end{aligned}$$

whereby the resultant power demand is calculated as $P_\text {d} = F_\text {t} v$.

Modeling of battery-packs for electric drivelines has been under continual development over the last few years. Individual cells in a battery-pack are subject to a heterogeneous spectrum of operating conditions and potential defectsbattspsonorispsmodel. Individual cell models are capable of capturing such effects to develop precise battery management and cooling strategies. However, such models are characterized with augmented complexity and are not suitable for optimization problems^34^. In this work, a second-order Thevenin (PNGV) model has been considered for the battery-pack, which is a common approach to provide a scalable and computationally-feasible optimization of electric powertrains without mitigating the fidelity and integrity of individual cell dynamics^35,36^. The implemented PNGV model proved an accuracy up to 98% compared to individual cell modeling in vehicular applications, where cell monitoring is not the main scope^37^.

**Figure 3:**
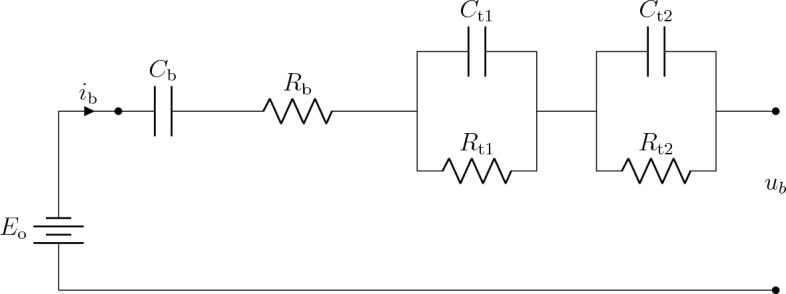
Equivalent circuit model of the battery based on second-order Thevenin model^41^.

The implemented second-order Thevenin (PNGV) model is illustrated in Fig. 3, where the battery voltage $u_\text {b}$ is calculated as

2 $$\begin{aligned} u_\text {b}&= E_\text {o} - i_\text {b} \ R_\text {b} - u_\text {t1} - u_\text {t2} - \frac{1}{C_\text {b}} \int _{t_\text {i}}^{t_\text {f}}i_\text {b}\ dt, \end{aligned}$$

considering $E_\text {0}$ as the open-circuit voltage. The polarization dynamics during battery charging and discharging are modelled through two series RC-Networks as

3 $$\begin{aligned} \begin{bmatrix} \dot{u}_\text {t1} \\ \dot{u}_\text {t2} \end{bmatrix} = \begin{bmatrix} - \frac{1}{R_\text {t1} C_\text {t1}} &{} 0 \\ 0 &{} - \frac{1}{R_\text {t2} C_\text {t2}} \end{bmatrix} \ \begin{bmatrix} {u}_\text {t1} \\ {u}_\text {t2} \end{bmatrix} + \ \begin{bmatrix} \frac{1}{C_\text {t1}} \\ \frac{1}{C_\text {t2}} \end{bmatrix} \ i_\text {b}, \end{aligned}$$

where $R_\text {t1, t2}$ and $C_\text {t1, t2}$ denote the equivalent Thevenin resistance and capacitance of each RC-network^38,39^. For the PMSM, a simplified mathematical model with a single input/multiple output (SIMO) state space model has been implemented as follows

4 $$\begin{aligned} \dot{x}_\text {m}&=A_\text {m} \ x_\text {m} + B_\text {m}\ u, \end{aligned}$$

5 $$\begin{aligned} y_\text {m}&= C_\text {m} \ x_\text {m} + D_\text {m} \ u, \end{aligned}$$

for

6 $$\begin{aligned} x_\text {m}&=\begin{bmatrix} \omega \quad i_\text {m} \end{bmatrix}^\text {T}, \ u=u_\text {m}(t), \end{aligned}$$

7 $$\begin{aligned} A_\text {m}&= \begin{bmatrix}-\frac{b_\text {m}}{J_\text {m}} &{} \frac{k_\text {eq}}{J_\text {m}}\\ \\ -\frac{k_\text {eq}}{L_\text {m}} &{} -\frac{R_\text {m}}{L_\text {m}} \end{bmatrix}, \ B_\text {m} = \begin{bmatrix} 0\\ \\ \frac{1}{L_\text {m}} \end{bmatrix}, \end{aligned}$$

8 $$\begin{aligned} \ C_\text {m}&=\begin{bmatrix}1&{}0\\ \\ 0&{}1\end{bmatrix}, \ \text {and} \ D_\text {m} = \begin{bmatrix}0\end{bmatrix}, \end{aligned}$$

whereby, the driveability is ensured based on a tuned observer-based PI-controller as discussed in^40^.

The considered driveline model in this contribution has been previously implemented for modeling and simulation of an inter-city e-bus with similar driveline characteristics^6,38^. The offered modularity in Simulink offers many advantages during parameter optimization, validation, and testing of the PMS algorithms within the driveline model when coupled with an experimental hardware-in-the-loop (HiL) and high-fidelity simulators for software-in-the-loop (SIL) simulation. We also use an in-house-developed optimization algorithm NSGA-II in MATLAB environment, which makes it more compatible to operate global variables, numerical constrains, and data logging on the same simulation platform.

**Figure 4:**
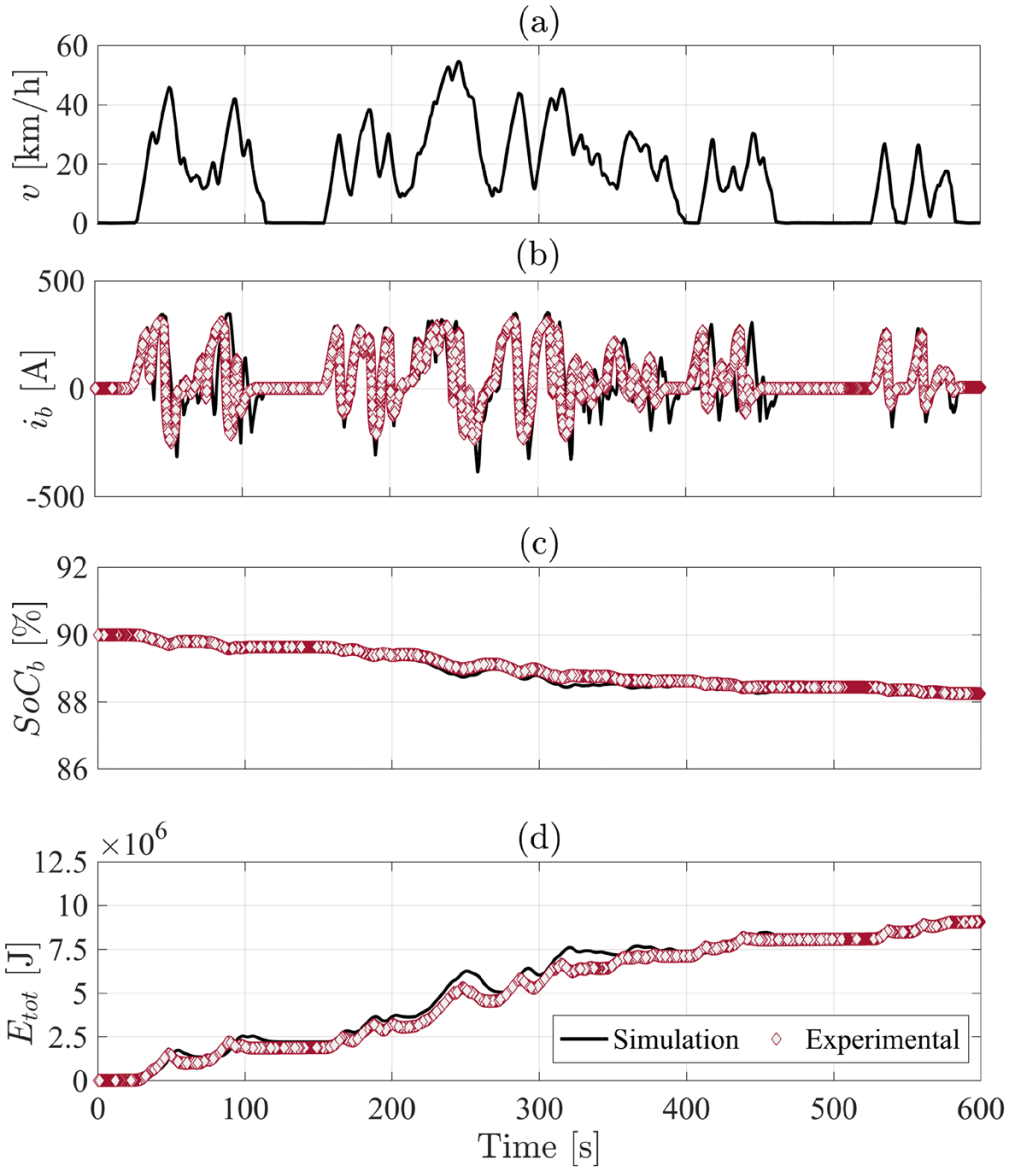
Simulation and experimental results of the electric bus during a standard energy consumption test over the WLTP driving cycle (according to UN-ECE Regulation No. 154).

The obtained simulation results of electric driveline have been comparatively evaluated against experimental measurements of standard charging/depletion tests, revealing a RMSE < 3%. Moreover, the experimental results of a standard energy consumption test of the investigated driveline (according to UN-ECE regulations no. 154) is compared to the outputs of vehicle model for the same test, revealing matched results with RMSE $=$ 0.1% for $SoC_\text {b}$, 4.4% for $E_\text {tot}$, and 7.8% for $i_\text {b}$ as shown in Fig. 4.

### Representative driving cycle and stops allocation

Gathering information about traveling speed and traffic density on specific routes is a costly process that involves the use of sensing instruments, on-board data-loggers, and trips organization. Besides, conventional methods of data gathering, i.e. car-chasing and on-board measurement, are subject to the influence of driver’s style and are characterized with limited coverage of daily driving conditions. These challenges stimulated many researchers to promote emerging surveillance systems that facilitates collection of crowd-sourced datasets^11^.

Web-based scraping of open-source traffic data has been increasingly implemented in research and industry to retrieve accurate and verified information about road speed and traffic conditions^6,11^. Besides, implementing crowd-sourced GPS-data has been proved to be particularly beneficial for the synthesis of representative driving cycles of open- and highway-type routes^5,42^.

**Figure 5:**
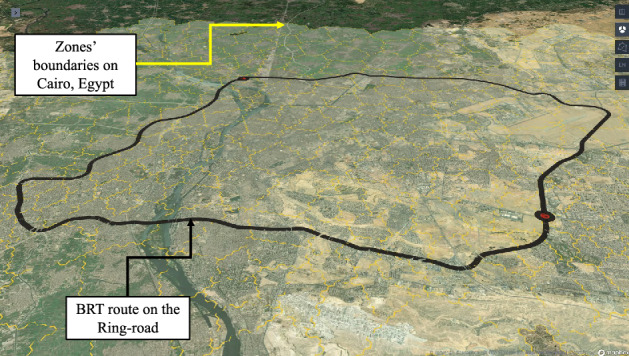
Geo-visualized representation of BRT route on the Ring-road through zone boundaries of Cairo, EG. (Visualized using Kepler.gl, an open-source geospatial web-based analysis tool for large-scale data sets^43^).

In this contribution, traffic data of the investigated route have been scraped on hourly basis over a total period of 6 months from July 1$\text {st}$ to December 31$\text {st}$ 2021. Gathered information include distance, speed, date and day-time. Traveling route of the E-BRT fleet is planned to circulate the urban and suburban areas of the city of Cairo, Egypt via the Ring-road over a total distance of nearly 79 km. The boundaries of city zones and traveling route of the E-BRT fleet are illustrated in Fig. 5.

**Figure 6:**
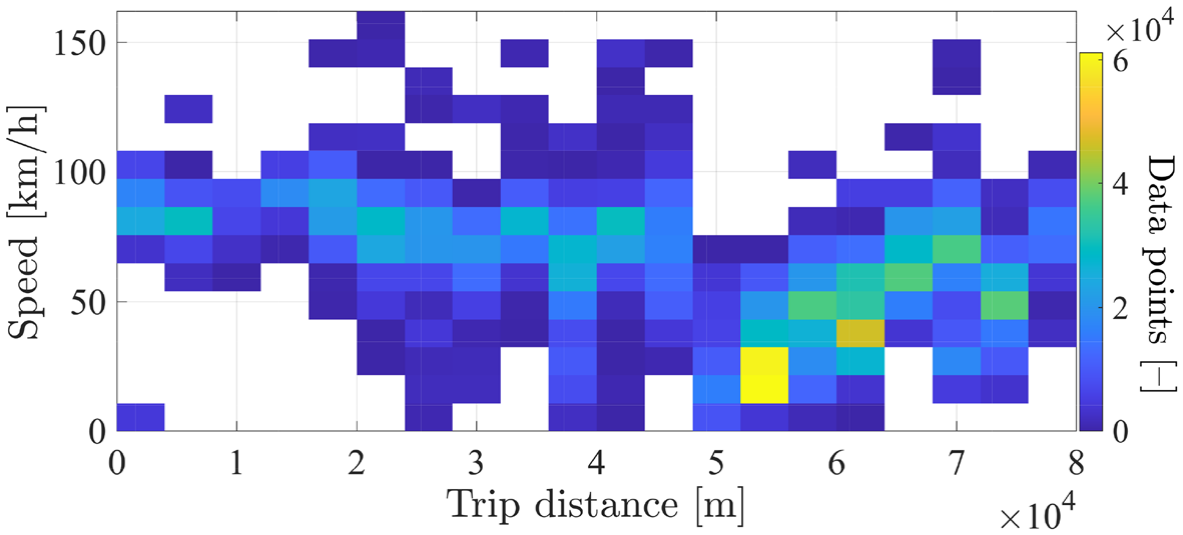
Histogram of scrapped data-points for speed-distance over the BRT route.

The histogram of speed values w.r.t. route distance is illustrated in Fig. 6. It can be perceived from Fig. 6 that the most frequent speed value for all route sections is nearly $\approx$ 70 to 80 km/h, knowing that the speed limit is 90 km/h for this route, except for the northern road exit (at trip distance x $\approx$ 55 km), where speed records less than 50 km/h have been densely captured at congested sections of the route.

**Figure 7:**
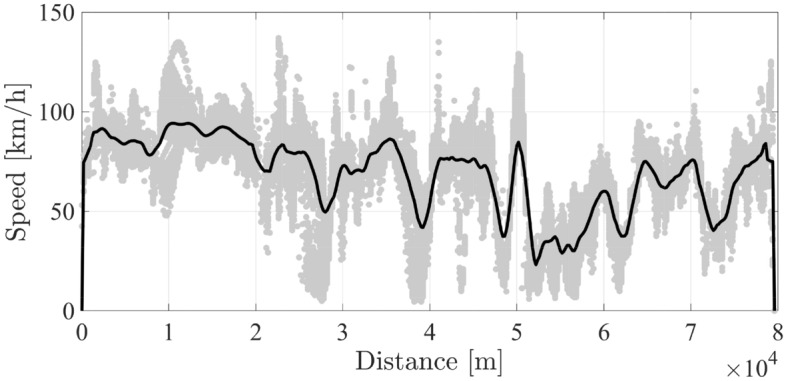
All scattered distance-based speed points with a single averaged one.

The histogramic representation of speed-distance data in Fig. 6 gives a clear insight into the homogeneity of traffic flow; which helps to identify representative speed profiles for the BRT trip. Considering the arithmetic mean of speed data-points over a non-overlapping horizon of 100 m, speed profile of the Ring-road can be constructed as illustrated in Fig. 7. Considered length of the non-overlapping horizon has been defined to reflect the average traffic flow dynamics and avoid the influence of individual outlying data-points for this particular case-study. For other types of routes, i.e. urban cycles or school-shuttles, the spatio-temporal characteristics of synthesized driving cycles should be set accordingly.

The optimality of power management of electric drivelines is significantly sensitive to the changes in driving speed profile, which influences the ability of such algorithms to yield desired control decisions^44^. Therefore, real driving cycles, in contrast to synthetic ones, are favorable to reflect actual trip conditions that influence the objective of implemented control strategy. To this aim, selection of data-point clusters to calculate the arithmetic mean of speed has been conducted based on cluster analysis of the whole dataset. In this context, multi-variant k-means clustering along with cluster analysis methods can be implemented to investigate the most significant number of clusters, that represent similar driving conditions distinctively^45^.

**Figure 8:**
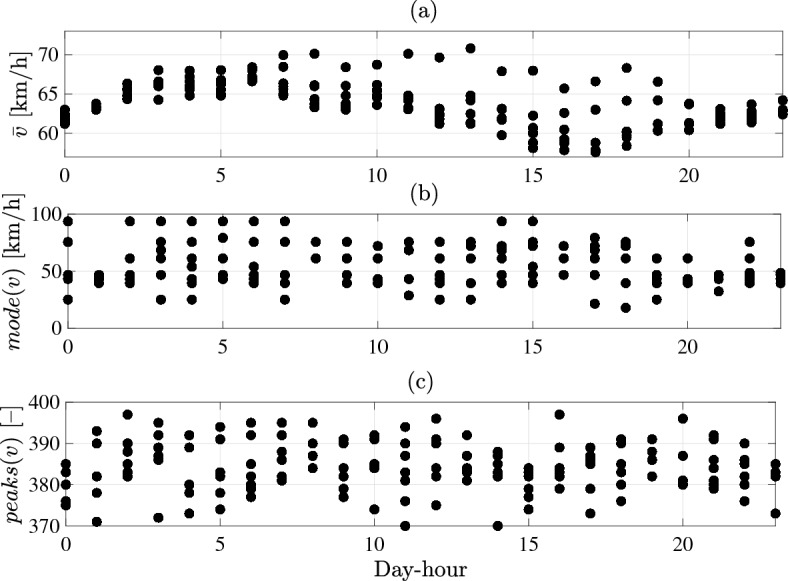
Statistical characteristics of gathered data of the representative driving cycle: (**a**) average speed ($\bar{v}$), (**b**) most frequent speed values *mode*(*v*), (**c**) number of peaks *peaks*(*v*).

Statistical characteristics of the spatio-temporal data are illustrated in Fig. 8. The average value of road speed ($\bar{v}$) and the most frequent value *mode*(*v*) of gathered data on hourly-basis are illustrated in Fig. 8a,b respectively. Total number of recorded speed peaks during each day-hour (*peaks*(*v*)) is shown in Fig. 8c, which gives an insight into the frequency of speed fluctuation and brake usage (driving aggressiveness) for the whole driving cycle. Different traffic conditions along day-hours can be perceived from Fig. 8, which reflects the need to define representative driving cycles according to respective conditions precisely. To this aim, gathered data on speed and acceleration (*v*(*x*, *t*), *a*(*x*, *t*)) with relevant statistical characteristics ($\bar{v}$, *mode*(*v*), *peaks*(*v*)) have been considered for the multi-variant k-means clustering to define relevant clusters of data-point in the representative driving cycles.

In this contribution, a set of data-points (time–speed–distance) for the investigated route are gathered over nearly 12 months to reflect the changing traffic conditions during the year. The implemented methodology to synthesize the representative driving cycle using the gathered dataset is mainly based on K-means clustering for a sliding horizon over the entire distance. This method has been developed and introduced in^6^ to establish a data-driven driving cycle for cross-country routes, to which accurate fleet sizing and stops’ allocation of urban transportation can be conducted. The same synthesizing procedures have been considered in this work to develop a distance-based driving cycle of the BRT route.

Initiated clusters of the gathered dataset $\{C_\text {1}, C_\text {2}, \ldots , C_\text {k}\}$ can be considered in a multi-dimensional space $\mathbb {R}$, such that

9 $$\begin{aligned}&\bigcup _{i=1}^{k} C_i = {\chi }\nonumber \\&\text {and} \quad \end{aligned}$$

10 $$\begin{aligned}&C_i \cap C_j = \varnothing \quad \qquad \forall {\left\{ \begin{array}{ll} i \ne j \quad \text {and}&{}\\ i,j \in [1, 2, \ldots , k] &{} \end{array}\right. } \end{aligned}$$

where $\chi \subset \mathbb {R}$ denotes the set of gathered data-points $x_\text {p}$, where $p=1,2,\ldots ,n$. Optimal clustering of $\chi$ has been conducted based on k-means++ algorithm^7,46^. For each data-point cluster $C_i$, $i=1,2,\ldots k$, the probability of finding suitable individual centroids $c_i$, at random data-points $x_\text {p}\,\in \,\chi$, for $p=1,2,\ldots ,n$, can be defined as

11 $$\begin{aligned} P_\text {c}&= \frac{d_\text {c}^2(x_\text {p},c_\text {i})}{\sum _{p=1}^{n} d_\text {c}^2(x_\text {p},c_\text {i})} \quad \forall {\left\{ \begin{array}{ll} i\in \{1, 2, \ldots , k\} \quad { \& } &{} \\ p\in \{1, 2, \ldots , n\}, &{} \end{array}\right. }{} & {} \end{aligned}$$

where $d_\text {c}$ denotes the distance between $c_\text {i}$ and arbitrary observations $x_\text {p}$. The points $c_1, c_2, \ldots , c_\text {k}$ that achieve minimal distance to the set of observations $X = \{ x_1, x_2,\ldots , x_\text {q} \}$, $q\leqq n$, are determined by iterative selection of arbitrary points $c_\text {i}$, $i=1, 2, \ldots , k$ in Eq. (11), such that $c_\text {i}\,\in \,\chi \,\subset \,\mathbb {R}$. Assignment of data-points of $\chi$ to respective clusters is conducted by solving the equation

12 $$\begin{aligned} x_\text {i}&\in C_{i_x} \qquad \text {if}\nonumber \\ i_\text {x}&= min. \{(x_\text {i} - c_\text {j} )\}, \qquad j=\{1,2,\ldots ,k\}, \end{aligned}$$

sequentially and iteratively to define the optimal set of clusters $C^*_1,$ $C^*_2$ $,\ldots ,$ $C^*_{k}$ with respective centroids

13 $$\begin{aligned} c^*_i&= \frac{1}{n_i} \sum _{x_\text {p} \in C_i}x_\text {p}, \qquad \forall {\left\{ \begin{array}{ll} i=\{1,2,\ldots ,k\} \text {and} &{} \\ p=\{1,2,\ldots ,n_i\}, &{} \end{array}\right. }{} & {} \end{aligned}$$

until $c_i^* = c_i, \ i= 1, 2,\ldots , k$^7^. The above-explained procedures are designed to ensure optimal clustering of $\chi$ considering a deterministic number of clusters *k*. However, selection of *k* is a non-trivial challenge in literature due to its influence on data compression and clustering accuracy^14^. Hence, evaluation of data clustering in Eqs. (9)–(13) has been analyzed considering achievable silhouette for $k= \{2:6\}$.

**Figure 9:**
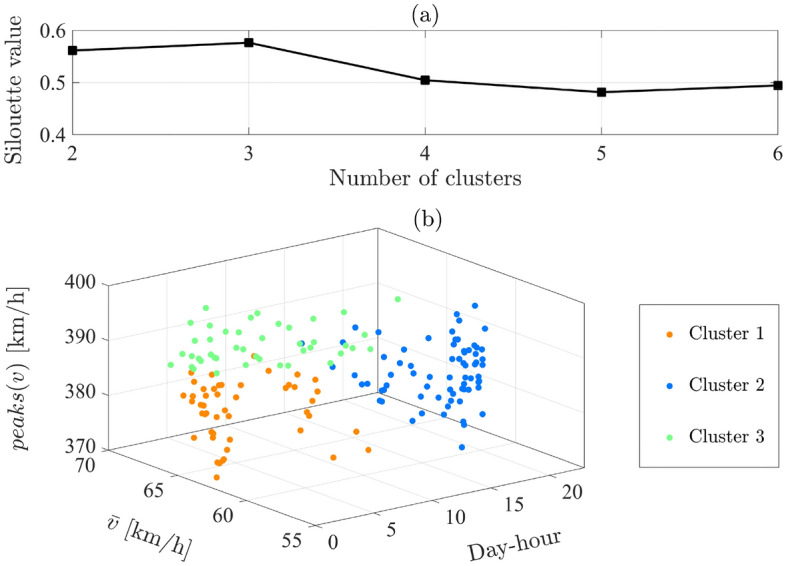
Finding optimal number of clusters of recognizable driving condition: (**a**) Silhouette value for different numbers of date clusters. (**b**) Clustered data-points of representative driving cycle considering an optimal value $k=3$.

Results of the silhouette analysis are shown in Fig. 9a for $k= \{2:6\}$. It can be perceived that the peak of achievable silhouette value is obtained for $k=3$, which implies an optimal balance between distinction and compression of data-points in clusters $C_1, C_2,$ and $C_3$. Clustered data-points in $\mathbb {R}$ are illustrated in Fig. 9b considering day-hours, average speed, and the number of speed-peaks. Representative driving cycles the investigated BRT-route can hence be generated for each cluster $C_1, C_2,$ and $C_3$, to be implemented for defining optimal power management strategy and recharge-scheduling of electrified buses as explained in the sequel.

## Optimal vehicular control and charge scheduling of the BRT fleet

Electrification of public transportation fleets has been made challenging by the inherited complexities of charge scheduling, infrastructural requirements, variable ridership capacity, and cost management. Estimation of ridership capacity has been addressed in^47^ by implemented a demand-aware Hopcroft–Krap algorithm to determine the required fleet dynamically considering temporal correlations between different districts in Wuhan, China and San Francisco, USA. The same problem has been investigated in^48^ by developing a novel infrastructure projection tool to estimate minimum required charging ports for a ride hailing fleet connecting 384 cities in the united states under multiple sources of input uncertainties. Genetic algorithm has been implemented to optimize charging stops of E-fleets considering limited capacity of the battery and concurrent use charging ports^49^.

The economical impact of on-road charging has been tackled considering optimal sizing and siting of supercapacitors’ swapping stops using discrete-integer bi-objective problem^50^. However, the computational viability of such algorithm has been validated only for small-dimension problems. The correlation between boost-charging, initial SoC of the battery, and charging capacity of the infrastructure has been studied for 60 different routes connecting a broad German territory, revealing the significant role of on-board control and route planning to sustain shorter travel time and efficient driving, compared to an inferior impact of increasing charging power at stations. Joint optimization has been conducted using mixed-integer programming in^51^. Despite the claimed improvement in infrastructural and operating cost, the proposed algorithm can be computationally expensive when deployed for large transportation networks.

The reviewed literature gives a clear insight into the significance of considering joint optimization of fleet sizing and charge scheduling of electric fleets to achieve near-optimal energy saving results. In this regard, evolutionary algorithms, i.e. GA, proved a particular competence to address multiple objectives of the problem and explore variable spaces efficiently at reduced computational requirements^52^. Furthermore, power management of E-fleets has been scarcely addressed in literature in combination with joint optimization procedures. Based thereon, simultaneous optimization of power management, fleet sizing, and charge scheduling using non-dominant sorting GA has been considered for the proposed work in this contribution.

Besides, it should be mentioned that representative driving cycles are mainly implemented to calculate battery discharge rates. Furthermore, it is required to determine the optimal capacity at the charging stop (number of fixed charging units) that ensures continual operation and minimizes the required number of vehicles in the fleet. In this context, the driving cycle is a deciding factor that implies the time of arrival of vehicles at the charging stop and hence increases the queuing and waiting time. To this aim, the synthesized driving cycle is also implemented to address these requirements in light of load profile of the grid.

For the optimal power management problem, two control variables have been considered: maximum battery current $\overline{i_b}$ and the upper limit of current rise/fall rate $\overline{\partial i_b / \partial t}$. These variables are accessible on the operational level of the investigated electric driveline and can be adjusted according to trip and driving conditions beforehand. The ability to recuperate regenerative energy and to ensure traction is directly influenced by defined $\overline{i_b}$; however, high C-rate values has an adverse impact on the depletion threshold and degradation rate of the battery^52^. On the other hand, inferior values of ${i_b}$ lead to adverse impact on driveability and entails longer trip duration^53^.

In this regard, driveability has been typically considered as an evaluation criterion of vehicular power management systems to ensure the fulfillment of required traction power^54,55^. The rationality of considering driveability into the multiple objective optimization is not conflicting with driving safety, yet it is pertinent avoid entrapment in trivial power management solutions^53^. Therefore, the cost function of the optimal control problem has been formulated as two objectives to represent contradictive corollaries of the above-mentioned control variables^56,57^. The optimal control problem can be defined as

### Problem 1

Optimal power management strategy considering on-board energy retention and driveability

14a $$\begin{aligned} {\textbf {min}} \qquad J&=\begin{bmatrix} O_{1}(u_1, v_1, t) \quad O_{2}(u_1, v_1, t) \end{bmatrix}^T,{} & {} \end{aligned}$$

14b $$\begin{aligned} \text {where}{} & {} {}&\end{aligned}$$

14c $$\begin{aligned} u_1&= [\overline{i_b} \quad \overline{\partial i_b / \partial t}],{} & {} \end{aligned}$$

14d $$\begin{aligned} v_1&= [i_b(t) \quad SoC_b(t) \quad d_{dc}(t)],{} & {} \end{aligned}$$

14e $$\begin{aligned} O_1&= \Delta SoC_b(t) \mid _{t=0}^{t=t_f} [\%],{} & {} \end{aligned}$$

14f $$\begin{aligned} O_2&= \int _{t_i=0}^{t=t_f} \frac{(P_d(t) - P_m(t))}{P_d(t)} dt \cdot 100 \ [\%],{} & {} \end{aligned}$$

14g $$\begin{aligned} \text {subject to}{} & {} {}&\nonumber \\ \underline{SoC_b}&\le SoC_b(t) \le \overline{SoC}_b,{} & {} \end{aligned}$$

14h $$\begin{aligned} i_b&\le \overline{i_b}, \quad \text {and}{} & {} \end{aligned}$$

14i $$\begin{aligned} {\partial i_b / \partial t}&\le \overline{\partial i_b / \partial t},{} & {} \end{aligned}$$

whereby the first objective $O_1$ represents the ability of optimized power management strategy to retain on-board energy without scarifying the driveability, which is represented in $O_2$, for the entire driving cycle. It should be emphasized that the driving cycle conditions, based on $d_{dc}$, is a main influencing variable to calculate the required battery power and state-of-charge dynamics in Problem 1.

It is noticeable that the driving cycle in Fig. 7 represents a single bus-commute in one direction of the BRT fleet. Time and energy saving of a single commute enhances the ability to conduct multiple commutes using the same vehicle before exceeding the lower limit of battery state of charge $\underline{SoC_b}$.

Non-dominant sorting genetic algorithm (NSGA-II) has been implemented to solve the optimization problem in Eq. (14), due to the proven ability of such evolutionary algorithm to tackle the complexity and non-linearity of optimization problems, handle co-dependent constraints, and explore the search space efficiently^58^. Optimization results of Eq. (14) using NSGA-II is discussed in conjunction with charge scheduling aspects in the sequel of “Analysis of results and discussion”.

Required bus headway on hourly basis for each route terminal $H (h, w_d, j_\text {s})$ can be defined as

15 $$\begin{aligned} H (h, w_d, j_\text {s})&= \frac{60}{F_r(h, w_d, j_\text {s})}, \ {\left\{ \begin{array}{ll} H \in \text {prime}(60)&{}\\ h = [1, 2, \ldots , 24]&{}\\ w_d = [1, 2, \ldots , 7]&{}\\ j_\text {s} = [1, 2, \ldots , 57]&{} \end{array}\right. }{} & {} \end{aligned}$$

where $F_r$ at predefined route stops can be calculated based on the minimum reciprocal frequency of vehicles $F_\text {r}^*$ (headway policy) as

16 $$\begin{aligned} F_r (h, w_d, J)&= max. [\frac{\vartheta (h, w_d, J)}{V_O(h, w_d, J)}, F_r^* (h, w_d, J)].{} & {} \end{aligned}$$

**Figure 10:**
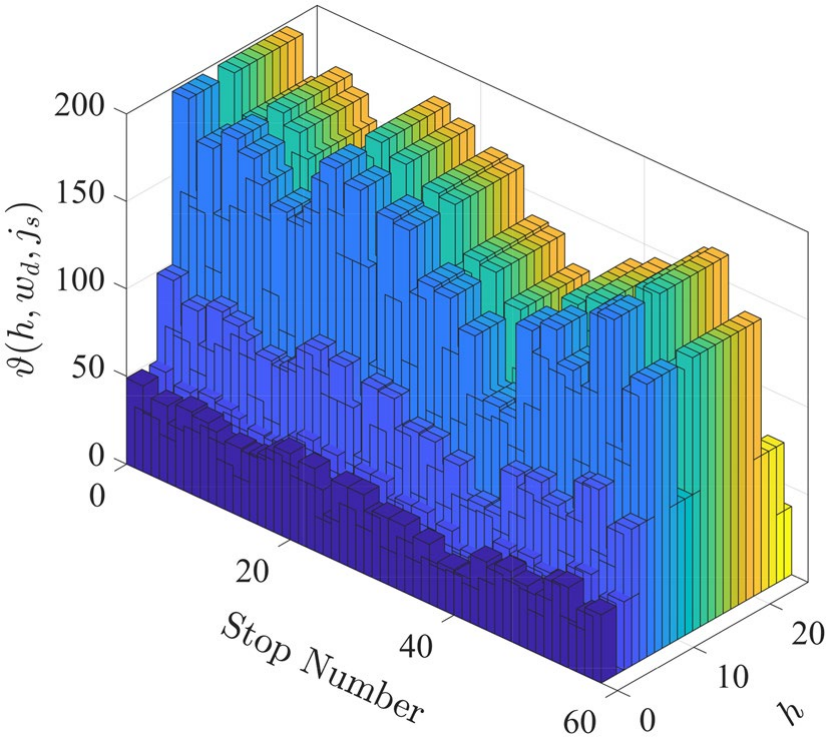
Expected number of passengers $\vartheta$ at predefined stops has been considered for normal weekdays and weekends.

The expected number of passengers at predefined stops has been considered for normal weekdays and weekends as illustrated in Fig. 10^59^.

Considering the predefined reciprocal frequency, number of stops, route characteristics, and representative driving cycle in Fig. 7, required fleet size $Z_f$ can be calculated as

17 $$\begin{aligned} Z_f&\geqq F_r(h, w_d) + V_{er}(h, w_d) + V_{ch}(h, w_d) + \cdots \nonumber \\&\qquad V_{q}(h, w_d), \quad \forall {\left\{ \begin{array}{ll} h = [1, 2, \ldots , 24]&{}\\ w_d = [1, 2, \ldots , 7]&{}\\ \end{array}\right. } \end{aligned}$$

such that

18 $$\begin{aligned} F_r(h, w_d)&\leqq V_{ra} + V_{ns} \qquad \text {and} \end{aligned}$$

19 $$\begin{aligned} V_{ch}(h, w_d)&\leqq N_{CB}, \end{aligned}$$

where $V_{ra}$ is the number of re-anchored vehicles that completed one successful commute followed by a time break $t_{br}$^60,61^.

**Figure 11:**
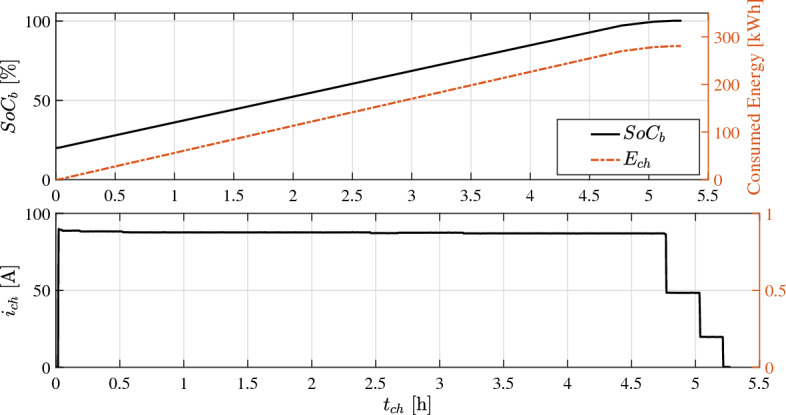
Experimental measurements for charging current, energy, and respective $SoC_b$ change during battery recharging in bus terminal.

Considering the optimal solution of Eq. 14 for $O_1^*(u_1, v_1, t)$, total number of commutes $\kappa _v$ for a single vehicle can be formulated as

20 $$\begin{aligned} \kappa _v = \frac{DoD_b}{O_1^*}, \end{aligned}$$

whereby the service time of a single vehicle can be calculated as

21 $$\begin{aligned} t_{st} = \kappa _v t_f + t_{br} + t_q + t_{ch}, \end{aligned}$$

where $t_\text {ch}$ is modeled based on validated experimental data in Fig. 11 as

22 $$\begin{aligned} t_{ch} = \frac{3600 \cdot Ah_b \ \eta _{ch}}{i_{ch}}. \end{aligned}$$

The energy consumption during battery recharging for $\varpi$-number of weeks can be formulated as

23 $$\begin{aligned} E_{ch}&= \varpi \sum _{h=1}^{24}\sum _{w_d=1}^{7} V_{ch}(h,w_d) Ah_b \frac{100 - SoC_\text {f}}{100},{} & {} \end{aligned}$$

which corresponds to an equivalent cost^62^

24 $$\begin{aligned} C_{eq}&= C_{e} E_{ch} \lambda _p, {\left\{ \begin{array}{ll} C_{e} = 1.6 \ \text {[E}\pounds /\text {kWh]}&{}\\ \lambda _p={\left\{ \begin{array}{ll} 0.923, &{}h \in \{1:9\},\\ 1, &{}h \in \{10:17,22:24\}, \\ 1.385, &{}h \in \{18:21\}. \end{array}\right. } \end{array}\right. }{} & {} \end{aligned}$$

**Figure 12:**
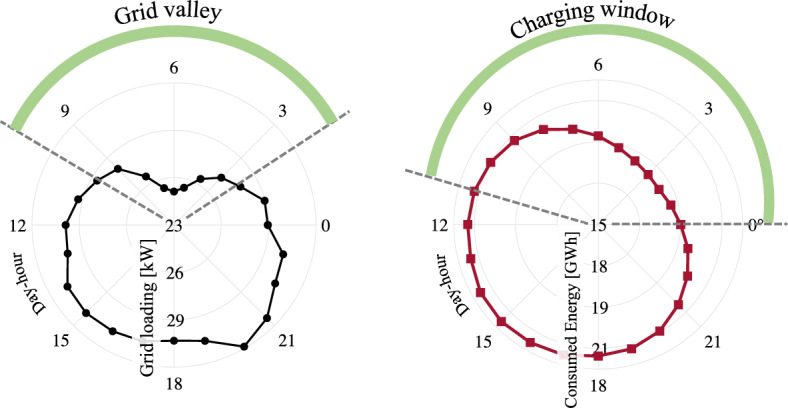
Average grid loading during day-hours and optimal time-window for fleet charging^63^.

It can be perceived from Eq. (23–24) that total cost of electric energy during battery recharging can be highly influenced by grid-loading as illustrated in Fig. 12. Therefore, defining suitable capacity of charging station $N_{CB}$ and contemporizing charging stops with off-peak hours has been considered in this contribution to minimize energy cost and queue time of vehicles’ recharging.

Joint optimization of fleet size, required number charging bays, and planned time-window for charging can be conducted considering the afore-explained aspects in Eqs. (15)–(24) comprehensively as

### Problem 2

Optimal fleet sizing and charge scheduling

25a $$\begin{aligned} {\textbf {min}} \quad J_f&=\begin{bmatrix} {O_{f1}(u_f, v_f) \ O_{f2}(u_f, v_f) \ O_{f3}(u_f, v_f)}\end{bmatrix}^T,{} & {} \end{aligned}$$

25b $$\begin{aligned} \text {where}{} & {} {}&\nonumber \\ u_f&= [{Z_f} \quad N_{CB} \quad h_{ch}],{} & {} \end{aligned}$$

25c $$\begin{aligned} v_f&= [V_{er} \quad V_{ch} \quad V_{q}],{} & {} \end{aligned}$$

25d $$\begin{aligned} O_{f1}&= Z_f [-],{} & {} \end{aligned}$$

25e $$\begin{aligned} O_{f2}&= \sum _{h=1}^{24}\sum _{w_d=1}^{7} C_{eq} \ [\text {E}\pounds ], \qquad \text {and}{} & {} \end{aligned}$$

25f $$\begin{aligned} O_{f3}&= F_r(h, w_d) - V_{ra} + V_{ns} - V_{ch} [-], \end{aligned}$$

whereto Eqs. (17)–(18) are considered as constraints of the optimization problem. The multi-objective optimization tackles initial cost (in $O_{f1}$, $O_{f2}$) and running cost in $O_{f3}$ by minimizing total charging cost of the fleet in accordance with grid loading profile. In this context, the driving cycle conditions are implicitly considered in Eq. (25) as a deciding factor to calculate the required energy for battery recharging $E_{ch}$ based on resulting drop of battery charge $SoC_{\text {f}}$. A comprehensive summary of the proposed methodology in this contribution is presented in Table 2.

**Table 2:** Optimal trip-oriented PMS, fleet sizing, and charge scheduling algorithm.

Algorithm: Optimal trip-oriented PMS, fleet sizing, and charge scheduling [$u^*$, $u_f^*$]		
1	**Inputs**	Route and driveline characteristics
		$\chi$, Eqs. (1)–(4)
2	**Initialize**	Representative driving cycle and significant speed profile clusters
		$v_{dc}$, $d_{dc}$, $C_1^*, C_2^*,\ldots , C_k^*$
3	**Solve**	Optimal route-oriented power management problem, Eq. (14)
	Using NSGA-II (10 levels for $u = [\overline{i_b} \quad \overline{\partial i_b / \partial t}]$ over 10 generations)	
		find $u^*$ achieving $J^* = \text {min.} J(u,v,t)$
		interim Output $u^* = [\overline{i_b}^* \quad \overline{\partial i_b / \partial t}^*]$,
4	**Initialize**	Headway requirements, Eq. (14)
		$$H, F_r, \vartheta , V_O$$,
5	**Solve**	Optimal fleet sizing and charge scheduling problem, Eq. (25)
	Using NSGA-II (10 levels for $u_f = [{Z_f} \quad N_{CB} \quad h_{ch}]$ over 10 generations )	
		find $u_f^*$ achieving $J_f^* = \text {min.} J_f(u_f,v_f)$
		interim Output $u_f^* = [{Z_f}^* \quad N^*_{CB} \quad h^*_{ch}]$,
**Outputs** optimal PMS, fleet size, and charge schedule $u^*$ and $u_f^*$		

## Analysis of results and discussion

**Figure 13:**
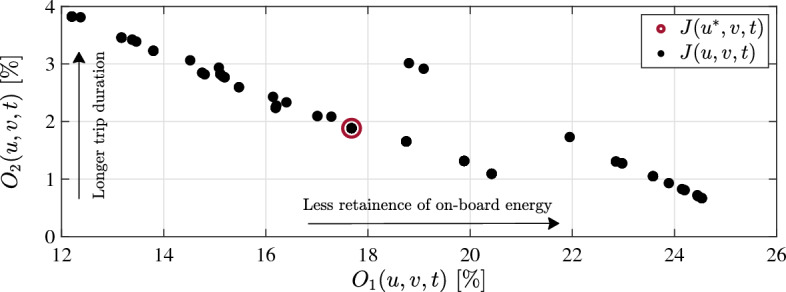
Pareto front and optimal solution of Problem 1.

Results of the optimal control problem in Eq. (14) considering representative driving cycle of the investigated route are presented in Fig. 13. The conflict between $O_1$ and $O_2$ can be perceived from illustrated results, which reflects the challenging task to achieve a trade-off balance between on-board charge retention and fulfilling driveability requirements. The optimal solution of Problem 1$J(u_1^*, v_1,t)$ has been yielded for $\Delta SoC_f =$ 17.68% at the cost of 1.9% mitigation of driveability.

**Figure 14:**
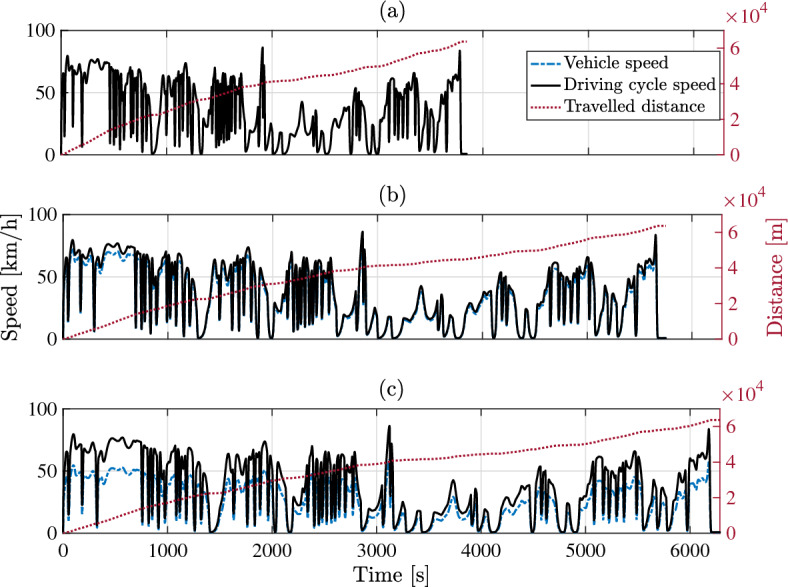
The impact of manipulating control variables in Problem 1 on trip duration. (**a**) Non-mitigated driving profile, (**b**) optimal solution, (**c**) arbitrary solution.

Mitigation of driveability has an adverse impact on trip duration, as actual vehicle speed becomes inferior to the reference speed of the representative driving cycle. The comparative illustration in Fig. 14 points outs the implicit contradiction between trip duration and retention of on-board energy in Problem 1. Shortest trip duration can be achieved at 44.5 min, considering ideal fulfillment of driveability (Fig. 14a). On the other side, arbitrary limitation of control variables, $[\overline{i_b} \ \overline{\partial i_b / \partial t}]$, leads to 135.3% increase in trip duration, i.a. 104.7 min (Fig. 14c). Conducting the optimal solution of Problem 1 puts forward the ability of route-oriented PMS to yield the above-mentioned results of $O_1$ and $O_2$ at trip duration of 77 min (Fig. 14b).

Resultant trip characteristics under the optimal solution of Problem 1 are implemented to explore the search space of Problem 2, considering trip duration, headway requirements, and allowable depth of discharge for the batteries. In this context, $t_f$ and $\Delta SoC_b$ have a direct influence on the number of vehicles ready to be re-anchored at main terminal of the route $V_{ra}(h)$ and increases the urge to assign new vehicles $V_{ns}(h)$. Consequently, number of reserve vehicles of the fleet is continuously influenced by the changes in $V_{ra}(h)$ and $V_{ns}(h)$ (see Eq. 25). Therefore, the results of Problem 2 in the sequel are simulated for a total of 52 weeks to count for accumulated impact of fleet operation, which may not be observed for limited number of operating weeks.

**Figure 15:**
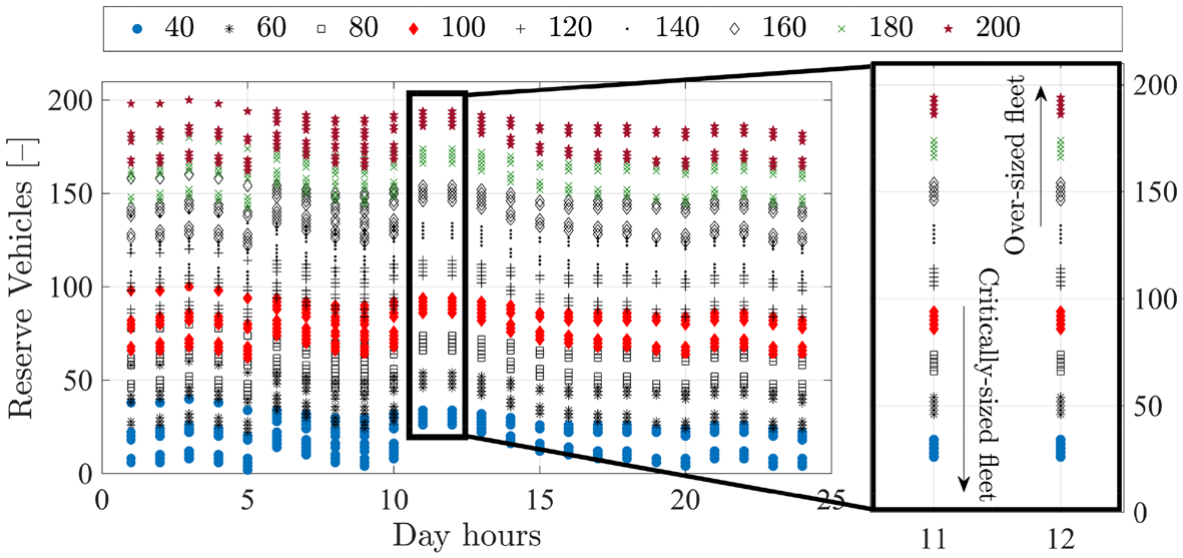
The impact of initial fleet size $Z_f$ on available number reserve vehicles on hourly basis.

The impact of initial fleet size on reserve vehicles is illustrated in Fig. 15, considering exemplified samples of $Z_f=[40:200]$. Illustrated results also reflect the influence of scheduling recharging durations at off-peak periods of the grid, resulting in a drop in available reserve vehicles at the end-time of the off-peak duration (9:00–10:00 am). Accordingly for downsized fleets ($Z_f=40$ as per example), critical cases of no reserve vehicles can occur at off-peak boundaries. Contrarily, a regular reserve of vehicles can be sustained on hourly-basis without being really assigned to trips.

The provided solution in this contribution is based on equivalent weighting between the objectives in Problems 1 and 2. To count for excessive unexpected capacity requirements, stochastic ridership functions can be considered into Problem 2 or by reducing the weighting of $O_{f1}$ (minimizing fleet size) to the favor of other objectives of $J_f$.

**Figure 16:**
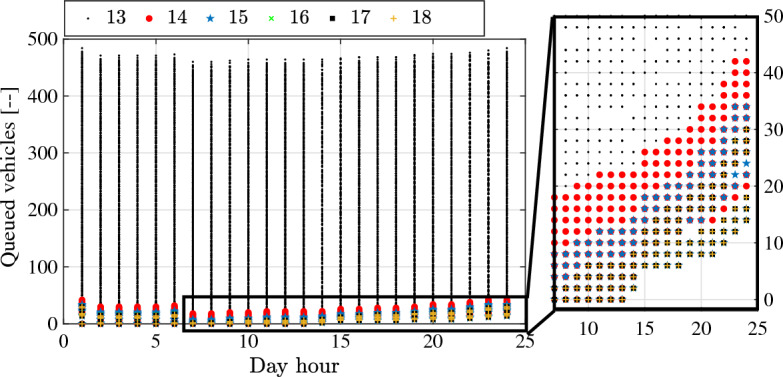
Total number of queued vehicle at recharging terminal on hourly basis considering different charging capacities $N_{CB}$.

On the other side, the influence of charging capacity $N_{CB}$ on total number of queued vehicles $V_q$ is illustrated in Fig. 16 for $N_{CB}=[13:18]$. Similar to the impact on reserve vehicles in Fig. 15, targeting optimal off-peak periods for vehicles’ recharging entails increasing queues at off-peak boundaries, with $V_q$ related to considered $N_{CB}$. Another important finding of Problem 2 is the marginal limit of $N_{CB}$ that stimulates an outbreak in $V_q$ regardless of day-hour, fleet-size, or off-peak periods. In this case, the marginal limit has been calculated as $N_{CB}=13$, which reflects the inability of the charging terminal with such capacity to fulfill operational requirements of the fleet robustly. On the other side, joint optimization of $N_{CB}$ and $Z_f$ is beneficial to avoid considering over-sized capacity of the charging terminal.

**Figure 17:**
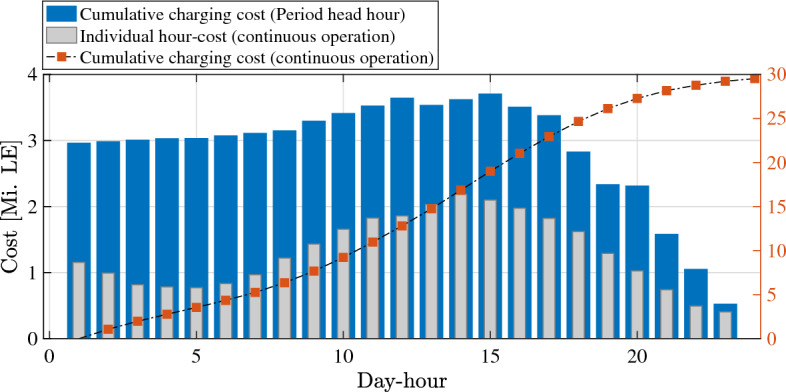
Annual cost of vehicles’ recharging considering continuous opening hours and off-peak periods.

Scheduling vehicle recharging round the clock is an ordinary strategy to avoid queues build up and reduce required fleet size. However, encountering over-priced energy tariffs at on-peak hours adds up to a significant running cost of the fleet annually. To point out the comparative evaluation of both strategy, annual cost of vehicles’ recharging is illustrated in Fig. 17, considering cumulative cost for round-the-clock strategy.

Annual charging cost considering off-peak hours (thick-blue columns) is illustrated in Fig. 17 for each day-hour individually, explicating the total cost of operating the charging terminal only for the upcoming 6 h daily. Such results reveal the significant potential to reduce annual energy cost for when synchronizing charging periods at off-peak hours. On the other side, annual energy cost considering round-the-clock operating hours is is illustrated for each day-hour individually (thin-gray columns), explicating the energy cost of on-charge vehicles during each day-hour. In this context, annual cost is calculated cumulatively for all day hour, which reveals the significant increase in energy cost compared to optimized off-peak charging strategy.

**Figure 18:**
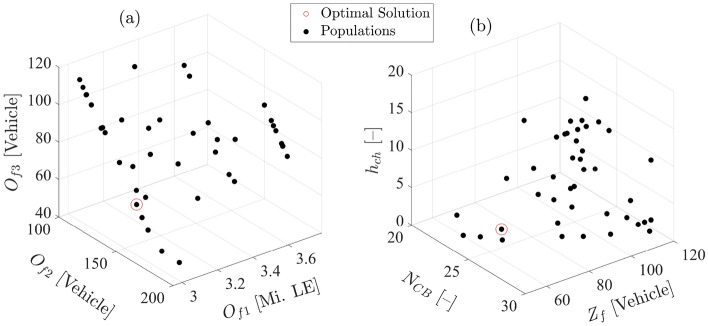
Pareto front and search space of control variables for Problem 2.

To sum up the results of joint optimization procedures, as summarized in Table 2, the search space of $u_f$ and the Pareto front of $J_f$ in Problem 2 are illustrated in Fig. 18. From a mathematical perspective, Fig. 18b reveals the ability to explore the search space comprising $Z_f$, $N_{CB}$, and $h_{ch}$ efficiently to proceed towards the optimal solution of the multi-objective cost function $J_f$. from another perspective, Fig. 18a shows the contradiction between $O_{f1}$, $O_{f2}$, and $O_{f3}$ to achieve a normalized balance between required fleet size, annual energy cost, and operation stability of the electrified fleet. The optimal solution (highlighted in red circles for $u_f*$ and $J_f^*$) has been yielded after 100 iterations of Problem 2.

It is important to mention that the conditions of driving cycle have a significant impact on charge scheduling and on-board control, which has been tackled in this work as follows: first, the characteristics of the driving cycle are determined considering the most influencing features (average speed, speed mode, and number peaks for all day hours). Consequently, optimal clustering of driving cycle segments has been conducted to enable a route-oriented optimization of the on-board control variables. Second, the solution of Problems 1 and 2 is derived to comprise the clustered driving cycle segments from previous step and hence is entirely adapted to any changes of the driving cycle when applied to other cases or routes. The simultaneous solving procedures of both problems (Table 2) is conducted to yield the optimal solution of both problems.

In light of these procedures, achieved results are comparatively evaluated in Table 3 against conventional methods of PMS and charge scheduling of electrified fleets. The conceptual difference between proposed methodology and conventional ones are summarized in the upper section of Table 3 as follows: first, representative speed profile of the investigated route has been developed and significant data-clusters within have been identified using cluster analysis. Second, route characteristics from first step have been implemented into Problem 2 to yield relevant power management strategies achieving optimal trade-off between on-board charge retention and driveability. Third, optimal fleet size, capacity of recharging terminal, and charge scheduling have been optimally defined to achieve minimal annual cost and fulfill operation requirements of the fleet.

**Table 3:** Comparative evaluation of fleet characteristics, energy efficiency, and ESS degradation of electrified fleets under different strategies.

	Conventional	Proposed solution	Improvement ^(†)^ [%]
Representative driving cycle Eq. (9)	X	$\checkmark$	–
Route oriented PMS Problem 1: Eq. (14)	X	${\checkmark }$	–
Fleet sizing & charge scheduling Problem 2: Eq. (25)	X	${\checkmark }$	–
Energy consumption [kWh/100 km]	215.59	173.93	**19.32**
Driveability [%]	100	98.1	**-1.9**
$\Delta SoC_f$ [%]	24.54	17.68	**27.95**
Fleet size [–]	120	71	**40.8**
Required $N_{CB}$ [–]	30	24	**20**
Annual charging cost [$\pounds E$]	3.79	2.96	**21.9**

^†^ Comparatively to the conventional solution.

In light of the aforementioned steps and operational constrains, an improvement of 28% in on-board charge retention has been achieved with vehicle driveability of 98.1%. Mitigation of abrupt accelerations and aggressive speed changes has contributed to reduce the average specific energy consumption to 174 [kWh/100 km], achieving an improvement of 19.3%. Besides, optimal number of required vehicles and recharging capacity for the electric fleet has been defined as 71 vehicle and 24 station to fulfill operational headway requirements efficiently. Optimal sizing of fleet and recharging requirements yielded an improvement of 40.8% and 20% respectively. Furthermore, scheduling vehicles recharging at off-peak periods enabled a reduction of 21.9% in annual energy cost. It should be mentioned that the achieved degree of optimality in this work is calculated in comparison to the baseline approaches. Implementation of global optimization methods is a potential improvement of the proposed approach.

The achieved results in this contribution put forward the significant impact of proposed methodology to achieve optimal driveability and operating cost of electrified fleets for urban public transportation. Hence, based on the outcomes of this research, major policy implications for electrified transportation can be pointed out as follows: first, electrification of BRT fleets in large metropolitans can be significantly beneficial for the environmental and economical footprint of public transportation. Second, the efficiency of electrified fleets is highly influenced by traveled driving cycles, allocated stops, and ridership. These variables should be considered carefully during strategic planning of fleet electrification. Third, simultaneous optimization of fleet size and charge scheduling in light of (analyzed route speed, stops, ridership, and infrastructural load) is crucial to ensure optimal energy and operational efficiency of the electrified fleet. Fourth, embracement of V2X data and power transfer technologies is an emerging endeavor to retain high operational efficiency and online adaptation of vehicular control (PMS) according to changing traffic and ridership conditions. This is a next-running extension of our research.

## Conclusion

This contribution proposed a novel approach to address the challenges of charge scheduling and power management of electric bus fleets, in light of uncertain driving conditions and fluctuating prices of electricity for battery charging. Representative driving cycle of the commuted route has been developed based on crowd-sourced information of traffic conditions; based on which, significant speed patterns has been identified using k-means clustering. Two multi-objective optimization problems have been formulated to investigate optimal control strategy and charge scheduling solutions achieving optimal energy efficiency, maximum retention of on-board energy, minimal mitigation of driveability, and minimum charging infrastructure and cost of the fleet.

The proposed approach has been implemented as a case-study for the electric bus rapid transit fleet of the city of Cairo, Egypt. Joint-optimization of the above-mentioned problems revealed a significant potential of the proposed approach to yield an improvement of 19% and 28% in energy consumption and retention of on-board energy accordingly, at the expense of less than 2% mitigation of driveability. Moreover, a reduction of 40.8%, 20%, and 21.9% in fleet size, required charging stations, and annual recharging cost respectively has been realized compared to the conventional fleet sizing and control methods.

In light of achieved results, the main innovations of this work can be summarized as follows: first, minimal energy consumption and overall cost of E-transportation have been comprehensively formulated, considering fleet-size, charge scheduling, and optimal vehicular control. Second, fluctuating electricity prices and daily traffic conditions have been integrated into the optimization scheme to yield a customized-optimal operating strategy of the fleet. Hence, an optimal sizing, operation, and charge scheduling of electrified fleets can be realized according to traffic conditions, ridership requirement, and infrastructural capabilities.

Next steps of this work are threefold: first, considering centrally-customized control strategies for each vehicle in the fleet individually to count for unscheduled changes in traffic conditions. Second, extending and merging the proposed concept for suburban fleets of the city, along with related electrification plans. Third, considering the respective CO_2_ footprint coordinated to vehicle charging at specific day-hours and at stations empowered by fossil-fuel or non-renewable energy.

## Data Availability

The datasets used and/or analyzed during the current study available from the corresponding author on reasonable request.

## List of symbols

*a*: Vehicle acceleration
$A_f$: Vehicle frontal area
$$Ah_b$$: Battery capacity
$b_m$: Viscous friction constant
*C*: Data point cluster
$$C_\text {b}$$: Fictive capacitance
$C_d$: Air-drag coefficient
$C_e$: Average electricity price
$C_{eq}$: Equivalent charging cost
$$C_{t1,2}$$: Equivalent capacitance
$DoD_b$: Depth of discharge
*d*: Distance
$d_c$: Distance between observations
$d_{dc}$: Driving cycle distance
$E_o$: Open-circuit voltage
$$E_{ch}$$: Charging energy
$E_{tot}$: Total depleted energy
$$F_t$$: Traction force
$F_r$: Reciprocal frequency of vehicles
*H*: Required bus headway
*h*: Day hours
*i*: Current
$$i_{ch}$$: Charging current
$$i_m$$: Motor current
*j*: Bus stop number
$J_m$: Rotational moment of inertia
$$k_{eq}$$: Electromotive constant
$$L_m$$: Equivalent inductance
$$m_v$$: Vehicle mass
$N_{CB}$: Number of charging bays
$$O_{f, 1}$$: Objective functions
$P_c$: Probability of centroid allocation
$R_b$: Battery int. resistance
$R_m$: Motor int. resistance
$$R_{t1, 2}$$: Equivalent resistance
*T*: Torque
*t*: Time
$$t_{br}$$: Time break
$$t_{ch}$$: Charging time
$$t_f$$: Final time
$$t_{st}$$: Service time
$$t_{q}$$: Queuing time
*u*: Voltage
$$u_f$$: Control vector
$$u_m$$: Motor voltage
$u_{t1, 2}$: Voltage across RC network
*v*: Velocity
$$V_O$$: Vehicle occupancy
$v_{dc}$: Driving cycle speed
$V_{er}$: No. of en-route vehicles
$V_{ch}$: No. of recharged vehicles
$V_{q}$: No. of queued vehicles
$V_{ns}$: No. of assigned vehicles
$V_{ra}$: No. of re-anchored vehicles
$$w_d$$: Weekdays
$x_p$: Single data points
$$Z_f$$: Fleet size
$$\eta$$: Efficiency
$\varpi$: Number of weeks
$\mu _r$: Rolling res. coeff.
$$\omega$$: Rotational speed
$$\rho _a$$: Air density
$$\theta$$: Road grade
$\vartheta$: Expected no. of passengers
